# Evaluation of a Game-Based Mechatronic Device for Rehabilitation of Hand-Arm Function in Children With Cerebral Palsy: Feasibility Randomized Controlled Trial

**DOI:** 10.2196/65358

**Published:** 2025-02-18

**Authors:** Mrudula Kanakapura Peramalaiah, Sanjay Tejraj Parmar, Nariman Sepehri, Saman Muthukumarana, Anuprita Kanitkar, Cherry Kit-Fong Hin, Tony Joseph Szturm

**Affiliations:** 1 SDM College of Physiotherapy Shri Dharmasthala Manjunatheshwara University Dharwad India; 2 Department of Mechanical Engineering, Price Faculty of Engineering University of Manitoba Winnipeg, MB Canada; 3 Department of Statistics University of Manitoba Winnipeg, MB Canada; 4 Department of Physical Therapy, College of Rehabilitation Sciences University of Manitoba Winnipeg, MB Canada

**Keywords:** cerebral palsy, computer game–assisted rehabilitation, manual dexterity, repetitive task practice, robotic manipulandum

## Abstract

**Background:**

Children with neurodevelopmental disorders, such as cerebral palsy (CP), often experience motor impairments in manual dexterity, which hinder daily tasks and social interactions. Traditional rehabilitation methods require repetitive task practice, which can be difficult for children to sustain due to low engagement. Game-based rehabilitation devices and robots offer a promising alternative by combining therapy with digital play, improving motivation and compliance. However, many systems fail to incorporate actual object manipulation, which is essential for motor learning through sensory feedback. To address this limitation, a low-cost, easy-to-use robotic manipulandum device (RMD) was developed. The RMD enables real-time object manipulation during gameplay while providing assistive force, allowing the practice of a wide range of manual dexterity skills beyond gross reaching. This system offers an engaging and effective rehabilitation approach to enhance hand function in children with CP.

**Objective:**

This study aimed to provide evidence for the feasibility and therapeutic value of the RMD game–based exercise program for children with CP.

**Methods:**

In total, 34 children with CP, aged 4 to 10 years, were randomly assigned to the experimental group (XG) or the control group (CG). The XG received a computer game–based exercise program using the RMD, focusing on object manipulation tasks, while the CG received task-specific training similar to constraint-induced movement therapy. Both groups received their respective therapy programs 3 times per week for 8 weeks. Semistructured interviews with parents and children, along with qualitative analysis, were conducted to evaluate their experiences with the exercise program. The following outcome measures were used: (1) the Peabody Developmental Motor Scale-2 (PDMS-2) grasping and visual-motor integration subtests and (2) the computer game–based upper extremity (CUE) assessment of manual dexterity.

**Results:**

No dropouts occurred during the 8-week program. Both groups showed significant improvements in the PDMS-2 subtests (*P*<.001) and the CUE assessment of manual dexterity, including success rates (tennis ball: *P*=.001; cone: *P*<.001; medicine ball: *P*=.001; and peanut ball: *P*<.001) and movement errors (tennis ball: *P*=.01; cone: *P*<.001; medicine ball: *P*=.04; and peanut ball: *P*<.001). The XG outperformed the CG, showing greater improvements in PDMS-2 grasping (*P*=.002) and visual-motor integration (*P*=.01). In the CUE assessment, the XG demonstrated higher success rates (medicine ball: *P*=.001 and peanut ball: *P*=.02) and fewer movement errors (cone: *P*<.001). Parents reported an increase in the children’s independence in daily tasks.

**Conclusions:**

This study demonstrates the feasibility, acceptability, and positive outcomes of the RMD game–based exercise program for improving hand function in children with CP. The findings support further research and development of computer game–assisted rehabilitation technologies.

**Trial Registration:**

Clinical Trials Registry - India CTRI/2021/07/034903; https://ctri.nic.in/Clinicaltrials/pmaindet2.php?EncHid=NTc4ODU

## Introduction

Children with cerebral palsy (CP) often experience motor impairments in the upper extremity, particularly in manual dexterity [1,2]. These motor deficits make it challenging for children to handle and manipulate objects, which are essential for many daily life activities, play, and social interactions [3,4]. Such impairments in fine motor control can limit their independence and hinder participation in a variety of activities that require precision and coordination [5].

Traditional therapeutic approaches, such as constraint-induced movement therapy [6-8] and hand-arm bimanual intensive therapy [9,10], have been shown to be effective in improving hand-arm function through repetitive task practice (RTP). These therapies focus on task-specific exercises and a high volume of practice [11,12]. However, maintaining children’s engagement in these therapies can be challenging due to the monotonous nature of the exercises, making it difficult to sustain motivation and adherence [13-15].

In recent years, there has been growing interest in integrating video games with rehabilitation programs to enhance engagement and motivation. These systems combine therapeutic exercises with digital gameplay, making the experience more enjoyable and encouraging adherence [16-18]. Devices like the Nintendo Wii, Kinect, Leap Motion, and inertial motion sensors have been used to track hand or arm movements and control digital avatars or objects in games [19-23]. Similarly, several studies have explored the effects of assistive robotic systems aimed at improving upper extremity function in children with CP. While many robots target the shoulder, elbow, and wrist range of motion [24-29], some focus on finger or thumb movements [30-32]. These systems use motion signals from robotic linkages (ie, body segment movements) to control digital avatars or manipulate a game paddle. However, both video game–based devices and robots often lack actual object manipulation, which provides critical tactile sensory information for detecting slip and maintaining stability, a key factor for effective motor learning [3,5,33-35].

To fill the gap in object manipulation, a game-based rehabilitation system using an inertial-based (IB) computer mouse was developed in previous studies, and its proof-of-principle was established [36-40]. The IB mouse is a miniature, wireless computer mouse that can be easily attached to any object, enabling interaction with video games through object manipulation [41]. This system allows children to practice manual dexterity tasks using real objects, with real-time feedback from video games. While effective for many children, this system does not provide movement assistance for those with limited active range of motion or poor motor control.

**Figure 1:**
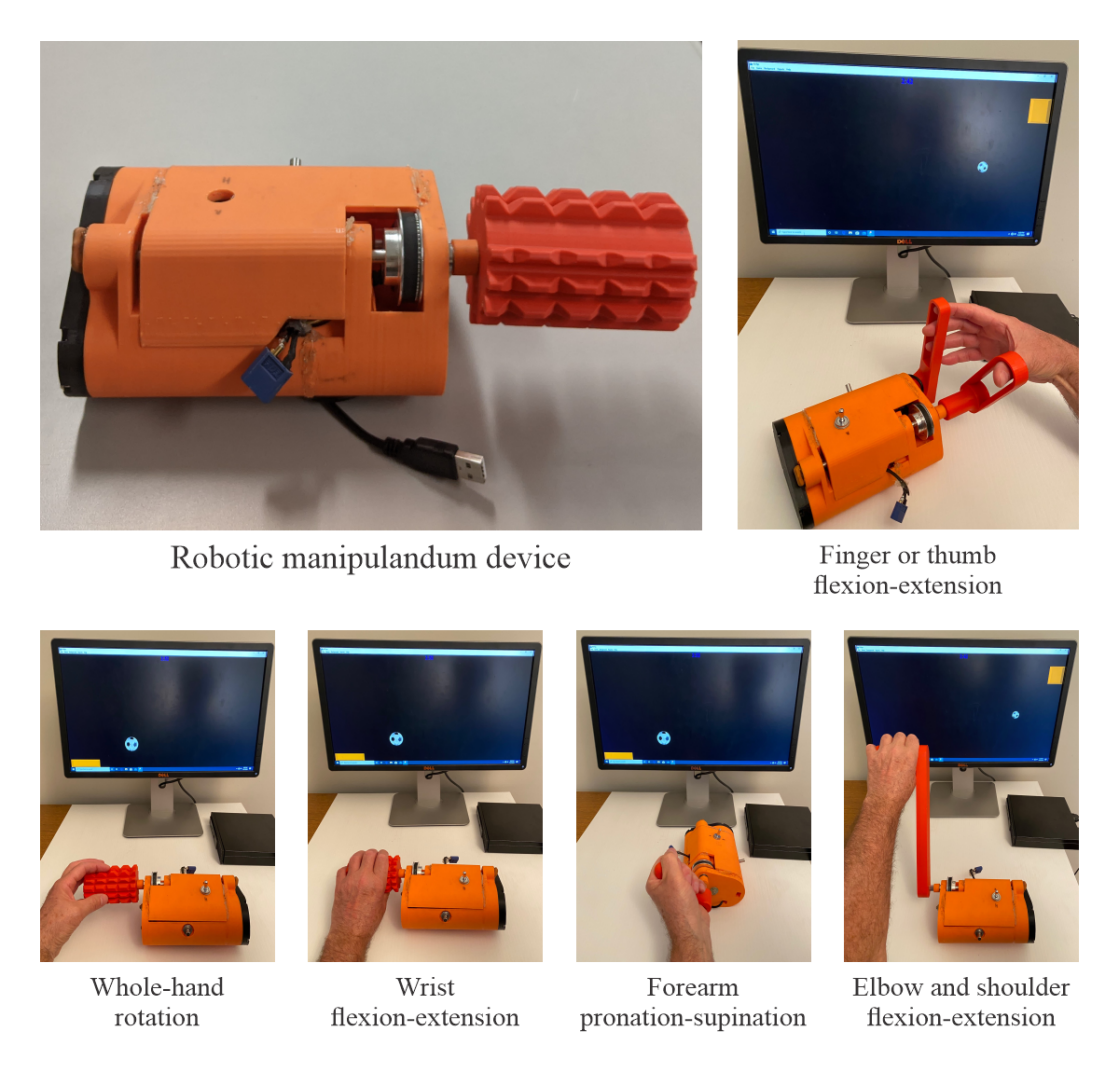
General view of the robotic manipulandum device and examples of various handles used in game-based rehabilitation for manual dexterity.

Taking the next step, a game-based robotic manipulandum device (RMD) was recently developed, which not only directly links object manipulation with computer games but also provides assistive force. As shown in Figure 1, the RMD consists of a small enclosure (15 cm by 10 cm by 8 cm) housing electronics, a motor, a controller, and a rotary drive shaft. A variety of 3D-printed handles of different shapes and sizes snap onto the RMD rotary drive shaft, as presented in Figure 1. These handles and their corresponding movements are designed to practice a broad range of manual dexterity skills involving (1) thumb or finger flexion-extension, (2) wrist flexion-extension and ulnar-radial deviation, (3) forearm pronation-supination, (4) elbow flexion-extension, and (5) shoulder flexion-extension and internal-external rotation. A microprocessor interfaces the RMD with computer games by reading the optical encoder on the shaft, which provides real-time angular position data to emulate mouse input. The angular position of the shaft (handle movement) is used to control the position and movement of the computer cursor or game sprite. This allows children to enjoy and engage with modern computer games, with varying levels of difficulty, speed, and accuracy, as part of a personalized exercise program. Many commercially available games also engage key visuomotor, perceptual, and cognitive skills. Specific therapeutic value can be derived from both the types of handles used (ie, different manipulation tasks) and the choice of computer games.

The RMD can provide unidirectional assistive force during gameplay, with the motor programmed to apply a constant force to the output shaft in a single direction. Both the magnitude and direction of this force are configurable. For many children with CP affecting the upper extremity, finger and wrist function may be more impaired in 1 direction of motion, for example, limited extension. This limitation can prevent children from positioning and opening the hand to grasp objects of varying diameters. The force assistance of the RMD allows the child to make larger game movement responses, making the system accessible to children with very limited active range of motion or poor motor control.

The purpose of this exploratory randomized controlled trial (RCT) is to enhance the development of the RMD and provide evidence of the feasibility of conducting a full-scale RCT. The objectives were to evaluate the usability, acceptance of therapeutic value, and treatment effect size of an exercise program using a game-based approach with the RMD in children with CP. The hypothesis is that an upper extremity exercise regimen using the experimental intervention will result in equal or greater improvements in hand-arm function compared to the usual outpatient physical therapy program. In parallel with this quantitative analysis, a qualitative analysis was conducted to explore the experiences of the children in the study and investigate both the difficulties with the exercises and using the technologies as well as the engagement and motivational value of the computer games.

## Methods

### Ethical Considerations

The study was conducted in accordance with international standards of Good Clinical Practice. Ethics approval was obtained from the institutional ethical committee at Shri Dharmasthala Manjunatheshwara University, Dharwad, Karnataka, India (approval number: 2021/Physiotherapy/MPT/07). The study was registered with the Clinical Trials Registry - India (CTRI/2021/07/034903) before the onset of participant enrollment. Both parents and children were provided with a clear explanation of the study’s objectives and methods. Written consent was acquired from the parents, while the children provided their assent to participate. All participants were informed of their right to opt out at any point without any repercussions. To ensure confidentiality, all data were anonymized, and no identifying information was published without explicit consent. No financial compensation was offered to the participants, and no images of the participants were included in the paper or any supplementary materials.

### Participants

Children diagnosed with CP were recruited for this single-blind randomized clinical trial with an active control arm. For the full CONSORT-EHEALTH (Consolidated Standards of Reporting Trials of Electronic and Mobile Health Applications and Online Telehealth) V1.6 checklist, see Multimedia Appendix 1. Participants were recruited from the Pediatric Physiotherapy Outpatient Department of SDM College of Medical Sciences & Hospital.

Inclusion criteria included (1) children with a confirmed medical diagnosis of CP, a neurodevelopmental disorder, by a medical practitioner or pediatrician; (2) age 4-10 years; (3) Gross Motor Function Classification System (GMFCS) from levels 1 to 3 [42]; (4) Manual Ability Classification System (MACS) from levels 1 to 3 [43]; (5) Modified Ashworth Scale indicating spasticity in finger and wrist flexors rated from 1 to 1+ [44]; and (6) the pediatric version of the Mini Mental State Examination scored 17 and above [45].

Exclusion criteria included (1) visual or auditory impairment, such that they cannot see and interact with the computer; (2) secondary orthopedic complications due to the neurodevelopment disorder like fixed deformities of upper extremities; (3) recent botulinum toxin therapy (less than 6 months); (4) recent surgical intervention of upper extremity; (5) moderate or severe spasticity, that is, Modified Ashworth score of grade 2 and higher; (6) cognitive impairment; and (7) uncontrolled seizures.

All parents and children were informed about the purpose and protocol of the study before enrollment; written informed consent was obtained from parents and assent from the children. Successfully screened participants were randomized to the experimental group (XG) or the control group (CG) by choosing 1 sealed opaque envelope containing a letter signifying the group assignment. A graduate student not involved in the study produced the envelopes, which contained a letter for either the XG or CG. The envelopes were thoroughly shuffled before each selection.

### Interventions

Participants attended 24 treatment sessions, 3 times a week for 8 weeks, at a university clinical rehabilitation research facility. Each session lasted 45 minutes.

#### Experimental Group

The experimental exercise program using the RMD was established based on the participants’ personal goals, the degree of their hemiparesis, and functional status. Figure 1 shows a variety of 3D-printed “therapy” handles of different shapes and sizes that snap onto the RMD rotary shaft. These were designed to practice a broad range of hand function skills. A typical session involved exercises with 4-5 different handles, several computer games, and assistive forces of different magnitudes. Each handle-game-force combination was practiced for 2- to 3-minute intervals and repeated 2-3 times. Different handles required different modes of manipulation, that is, game movement responses produced by thumb, finger, wrist, forearm or elbow, and shoulder movements. Task demands were adjusted by changing mouse sensitivity, movement range, etc. Different games were also selected to adjust movement speed and precision. Progression was achieved by using a variety of commercial computer video games. Many readily available and inexpensive computer video games have therapeutic value. For example, the commercial internet website “Big Fish” games contains hundreds of arcade-style computer games in several genres, many of which are appropriate for the game-based RMD exercise program. In addition to speed, target, and accuracy movement requirements, these games include several cognitive elements, such as games with different types of distractor objects to avoid, matching activities, and those that require ordering objects. See Multimedia Appendix 2 for several examples of commercial computer games from the Big Fish website, which were used in this study. These computer video games offer sufficient diversity to appeal to a broad range of individual preferences. Progressing the difficulty levels regularly and introducing different computer video games to sustain the challenge and provide new experiences will facilitate the psychological feedback required to maintain interest and participation.

#### Control Group

This included a goal-directed, physical therapy program. The protocols were based on RTP-based principles of modified constraint-induced movement therapy and hand-arm bimanual intensive therapy for fine and gross motor skills. Training tasks were individualized for every child according to their level of impairment and preset goals. Individualized exercises such as reaching for objects, removing rings and putting them back, ball throwing (under and overarm), turning a doorknob, clay activities, picking marbles from sand, putting pellets, rings, and pegs into sockets, opening bottle caps, manipulating objects such as a toy car, crumbling paper, hand-arm bimanual activities such as holding the ball with both hands and transferring, rolling bolster, writing, and self-feeding were chosen as part of the conventional protocol in a one-on-one therapy session.

### Quantitative Analysis

The following outcome measures were obtained prior to and following the 8-week intervention.

#### Computer Game–Based Upper Extremity Assessment of Manual Dexterity

A miniature, wireless IB computer mouse (Therapy Mouse, Mobility Research) was secured to 4 test objects with different physical properties and functional demands. When the IB mouse is attached to a “test” object, the manipulation of the object is used to control the motion of a game paddle of a purpose-built rehabilitation assessment application. For a full description of the computer game–based upper extremity (CUE) assessment, data recording, and analysis, see references [5,29,32].

**Figure 2:**
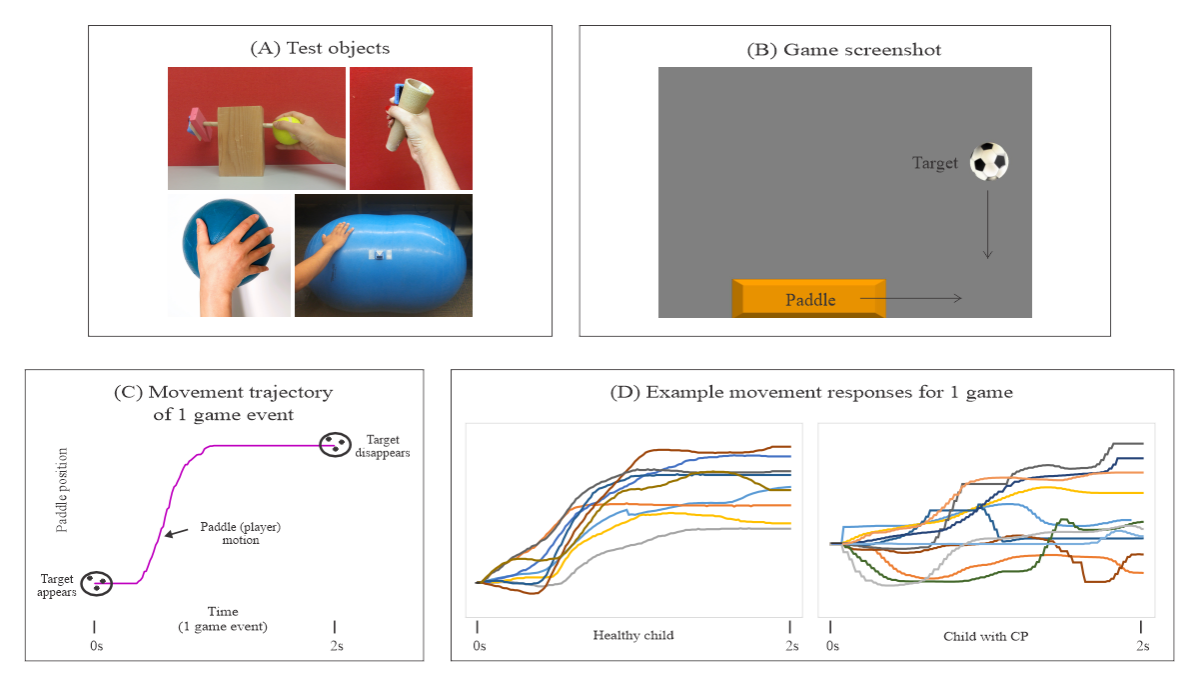
Illustration of the computer game–based upper extremity assessment setup and data recording. Panel (A) displays 4 pictures of different test objects, each instrumented with an inertial mouse. Panel (B) shows a screenshot of the computer game–based upper extremity assessment game, featuring the target object (computer-controlled) and the game paddle (controlled by the rotation of the tennis ball). Panel (C) presents a single game movement trajectory (game paddle coordinates) for 1 game event, from target appearance (time 0) to target disappearance (time 2 seconds). Panel (D) depicts overlay plots of segmented and sorted game movement trajectories for a 60-second game trial. The plots illustrate the rightward rotation of the tennis ball, with the left plot showing data from a healthy child and the right plot representing data from a child participating in this study. CP: cerebral palsy.

Figure 2A presents the features of the four test objects:

1. Tennis ball rotation: It is a simple crafted object consisting of a wooden block and dowel. A hole was drilled in the wooden block and the wooden dowel was inserted. The IB mouse was attached to one end of the wooden dowel, and the other end was inserted into a tennis ball. Children grasped the tennis ball between their thumb and fingers and rotated the ball leftward and rightward to move the game paddle during gameplay.
2. Cone: The children grasped the cone and used pronation and supination to move the game paddle during gameplay.
3. Medicine ball (20 cm diameter): The children placed their hands on top of the medicine ball and used ulnar and radial deviation to rotate the ball and move the game paddle during gameplay.
4. Peanut ball: The children placed their hands on top of the peanut ball and moved it forward and backward using a combination of elbow flexion-extension and shoulder flexion-extension during gameplay.

A custom-designed rehabilitation assessment game, the RTP game software developed by the University of Manitoba, was used to evaluate the object manipulation tasks. The children were seated at a table with adjustable height. The test objects were placed on the table at a comfortable reaching distance. A standard computer monitor was used to display the RTP computer game tasks. The monitor was placed 1 meter in front of the children at eye level.

As illustrated in Figure 2B, a game target object (soccer ball) appears at the top edge of the display and moves to the bottom edge of the display. When the target object reaches the bottom, it disappears. The children were instructed to rotate the IB mouse on each of the 4 test objects to control the game paddle and catch the moving target object. Each game event from target appearance to its disappearance took 2 seconds. Each game trial was 60 seconds in duration; therefore, 30 game movement responses were obtained for analysis, with half of the game movement responses in each direction. The position of each successive target appearance was randomized. The RTP game software logs the coordinates of the game paddle (object rotation) and target game objects at 100 Hz for offline analysis.

Figure 2C presents the trajectory of a typical single game movement response. Figure 2D presents overlay plots of all game movement responses in 1 direction for 1 game trial. In this illustration, when using the cone as the game controller in the right hand, upward traces (rightward paddle movements) would represent supination.

The following outcome measures of structure and function were derived from the recorded game movement responses of each test object manipulation task:

- Success rate: It is the percentage of the total number of target objects that were caught in 1 game trial.
- Movement error: When a target is missed, the magnitude of the error (distance between paddle and target position) is calculated. The average value for all misses is then computed as the movement error. Units are the percentage of screen width.

The CUE assessment has shown moderate to high test-retest reliability [32].

#### Two Subtests of the Peabody Developmental Motor Scale-2

The two subsets of the Peabody Developmental Motor Scale-2 (PDMS-2) are as follows: (1) grasping subtest includes 26 items that measure a child’s ability to use their hands and (2) visual-motor integration (VMI) subtest includes 72 items measuring a child’s ability to use visual perceptual skills. Both the PDMS-2 grasping and VMI subtest scores have shown high test-retest reliability and good construct validity [46,47].

### Qualitative Analysis

Upon completion of the 8-week exercise program, parents and children enrolled in the XG were invited to participate in an interview. The following open-ended questions were asked of all participants, and their responses were recorded: (1) When you agreed to participate, how did you hope your child would benefit from the therapy program? (2) What did you like about the therapy program? (3) What was challenging about implementing the therapy program for your child and you? (4) What did you think about the exercises or games or RMD your child was asked to perform? (5) How did your child respond to the exercises or games? Was there any exercise that your child did not seem to enjoy? (6) Would you want your child to continue with the same type of therapy program? Why or why not?

Parents and children who participated in the interviews were requested to share their ideas, thoughts, opinions, and personal experiences. The inductive analytical framework of interpretive description was used for qualitative analysis [17,48].

A staff physiotherapist who was blinded to the intervention conducted all the interviews. Interviews were conducted in the local preferred language (Kannada or English), and audio recordings were later transcribed into a written format. Transcripts were translated professionally, and then one researcher developed the thematic system by coding and categorizing the written transcripts of each interview. A second researcher reviewed the categorized data, identified any additional unique responses, and finalized the codes organized into final themes and subthemes.

### Statistical Analysis

Descriptive statistics, including means, 95% CIs, and percentages, were used to summarize demographic variables and outcome measures. The Shapiro-Wilk test was used to assess the normality of the data [49]. Comparisons between groups were evaluated using the independent samples 2-tailed *t* test for continuous variables and the chi-square test for categorical variables. To analyze the effects of time (pre- to postintervention), group (XG and CG), and the time×group interaction on the CUE assessment, PDMS-2 grasping, and VMI, a mixed model ANOVA with repeated measures was used. Effect sizes for the ANOVA were computed using η^2^, with the thresholds for small, moderate, and large effects defined as 0.01, 0.06, and ≥0.14, respectively, following Cohen guidelines [50]. All statistical analyses were conducted using SPSS (version 24; IBM Corp).

## Results

### Overview

**Figure 3:**
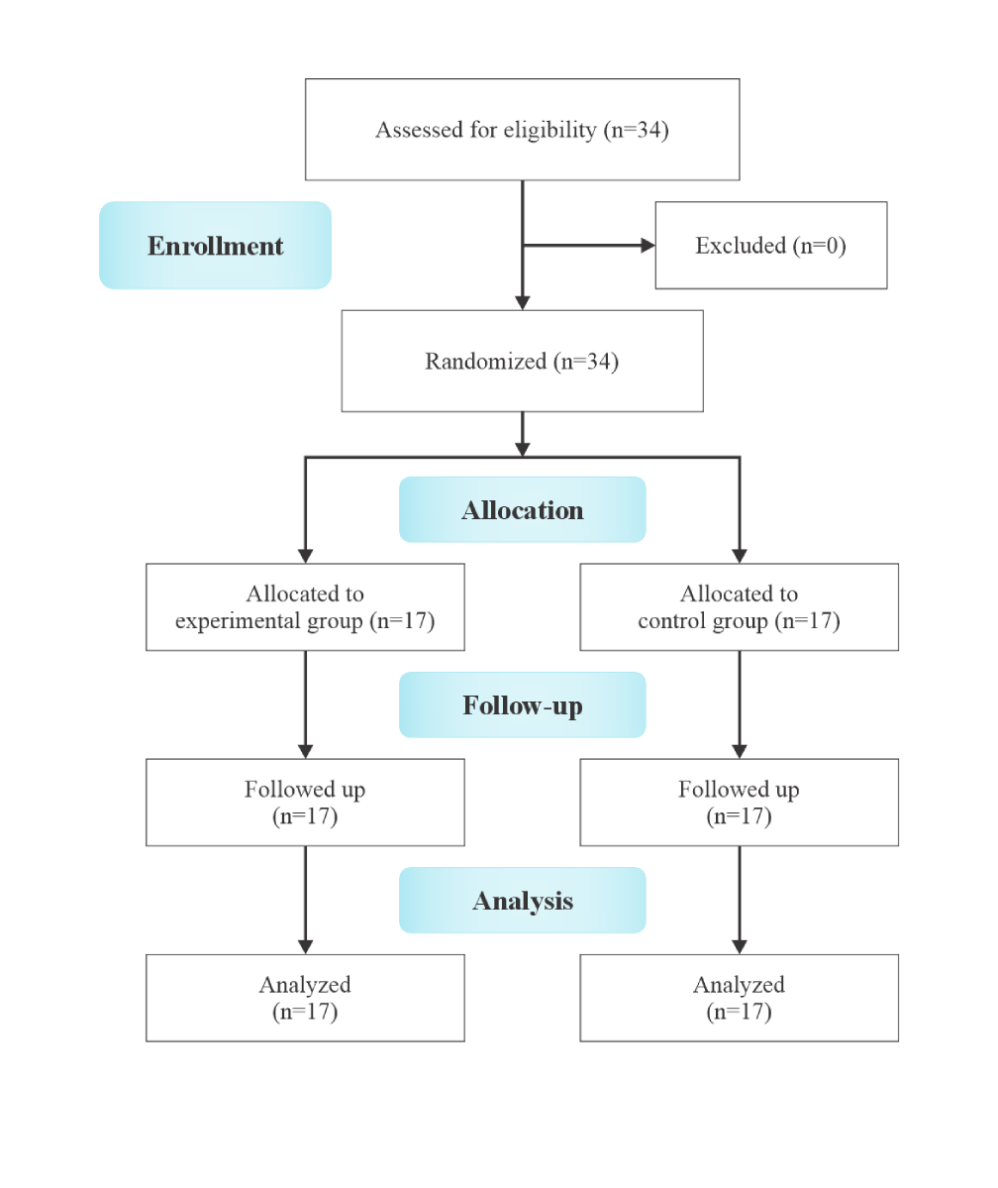
CONSORT (Consolidated Standards of Reporting Trials) diagram for the randomized clinical trial.

The recruitment target of 34 children was achieved within 8 months. All enrolled children completed both pre- and postassessments and attended all exercise sessions, resulting in a compliance rate of 100%. There were no adverse events or technological issues reported during the exercise programs. These findings demonstrate excellent feasibility. Figure 3 presents the CONSORT (Consolidated Standards of Reporting Trials) diagram, illustrating the participant flow throughout the study.

Table 1 presents demographic and clinical data categorized by group. The data were normally distributed, and there were no significant differences between groups at baseline in terms of age, sex, height, weight, GMFCS or MACS level, or treated hand. The majority of children were classified as GMFCS level II and MACS level II. The XG exhibited significantly higher scores in PDMS-2 grasping and VMI compared to the CG.

**Table 1:** Demographic and clinical data.

		XG^a^ (n=17)	CG^b^ (n=17)	*P* value^c^	
Age (years), mean (SD)		7.2 (2.8)	8.4 (2.5)	.21	
Height (cm), mean (SD)		112.2 (14.7)	120.1 (18.9)	.19	
Weight (kg), mean (SD)		19.6 (5.8)	23.5 (10.4)	.18	
**Sex, n (%)**					>.99
	Female	8 (47)	8 (47)		
	Male	9 (53)	9 (3)		
**Treated hand, n (%)**					.28
	Left	3 (18)	2 (12)		
	Right	12 (71)	15 (88)		
	Left and right	2 (12)	0 (0)		
**GMFCS^d^, n (%)**					.42
	Level I	2 (12)	5 (29)		
	Level II	11 (65)	8 (47)		
	Level III	4 (23)	4 (23)		
**MACS^e^, n (%)**					.21
	Level I	6 (35)	6 (35)		
	Level II	9 (53)	5 (29)		
	Level III	2 (12)	6 (35)		
PDMS-2^f^ grasping, mean (SD)		45.7 (5.2)	39.5 (7.3)	.01	
PDMS-2 VMI^g^, mean (SD)		115.7 (19.4)	87.1 (19.3)	.001	

^a^XG: experimental group.

^b^CG: control group.

^c^Statistical significance was assessed using the independent samples 2-tailed *t* test for continuous variables (age, height, weight, PDMS-2 grasping, and PDMS-2 VMI) and the chi-square test for categorical variables (sex, treated hand, GMFCS, and MACS) to compare differences between the XG and the CG.

^d^GMFCS: Gross Motor Function Classification System.

^e^MACS: Manual Ability Classification System.

^f^PDMS-2: Peabody Developmental Motor Scale-2.

^g^VMI: visual-motor integration.

**Figure 4:**
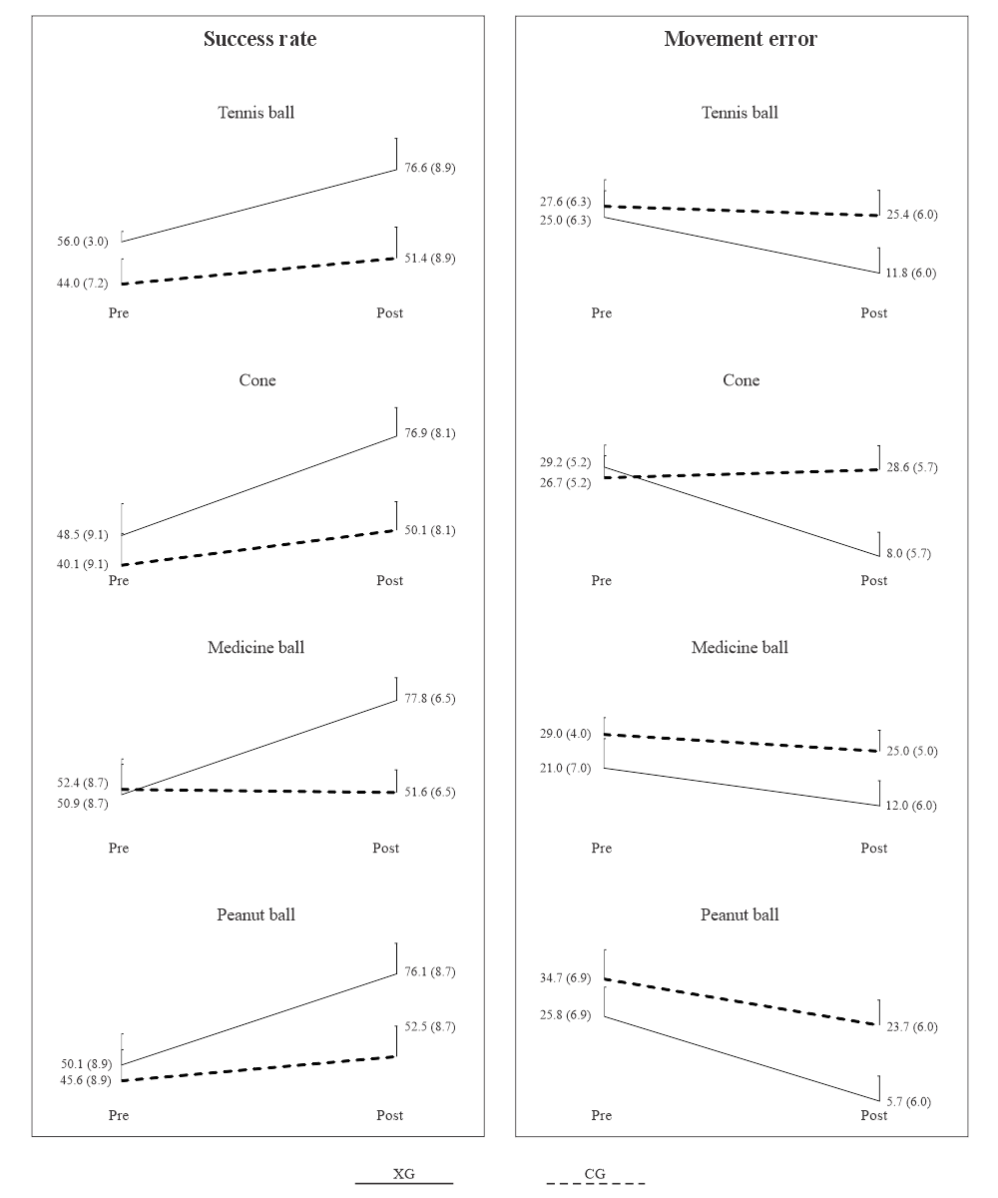
Group means and 95% CIs of success rate and movement error for the 4 test objects before and after the intervention. Solid line indicates XG, and dashed line indicates CG. CG: control group; XG: experimental group.

Tables 2 and 3 present the ANOVA results for success rate and movement error, respectively, analyzing the effects of time, group, and the time×group interaction. Figure 4 illustrates line plots of group means and 95% CIs before and after the intervention for success rate and movement error. Significant time and group effects were observed with moderate to large effect sizes across all 4 object manipulation tasks. Figure 4 highlights greater improvements in success rate and movement error for the XG compared to the CG, particularly evidenced by significant interaction terms in 3 of 4 object manipulation tasks for success rate and 2 of 4 tasks for movement error.

**Table 2:** ANOVA results for time, group, and the time×group interaction on success rate.

Test objects	Time of the assessment (within-group)				XG^a^ vs CG^b^ (between-group)				Time×group		
	*F* test (*df*=1)	*P* value	η^2^	*F* test (*df*=1)		*P* value	η^2^	*F* test (*df*=1)		*P* value	η^2^
Tennis ball	12.66	.001	0.28	30.77		<.001	0.49	1.50		.23	0.05
Cone	17.11	<.001	0.35	21.73		<.001	0.40	3.93		.056	0.11
Medicine ball	12.07	.001	0.27	10.74		.003	0.25	13.74		.001	0.30
Peanut ball	18.13	<.001	0.36	8.85		.006	0.22	6.11		.02	0.16

^a^XG: experimental group.

^b^CG: control group.

**Table 3:** ANOVA results for time, group, and the time×group interaction on movement error.

Test objects	Time of the assessment (within-group)				XG^a^ vs CG^b^ (between-group)				Time×group		
	*F* test (*df*=1)	*P* value	η^2^	*F* test (*df*=1)		*P* value	η^2^	*F* test (*df*=1)		*P* value	η^2^
Tennis ball	7.11	.01	0.18	6.68		.02	0.17	3.62		.07	0.10
Cone	18.42	<.001	0.37	8.62		.006	0.21	26.40		<.001	0.45
Medicine ball	4.49	.04	0.12	63.02		<.001	0.66	0.10		.75	0.003
Peanut ball	24.83	<.001	0.44	17.23		<.001	0.35	2.16		.15	0.06

^a^XG: experimental group.

^b^CG: control group.

**Figure 5:**
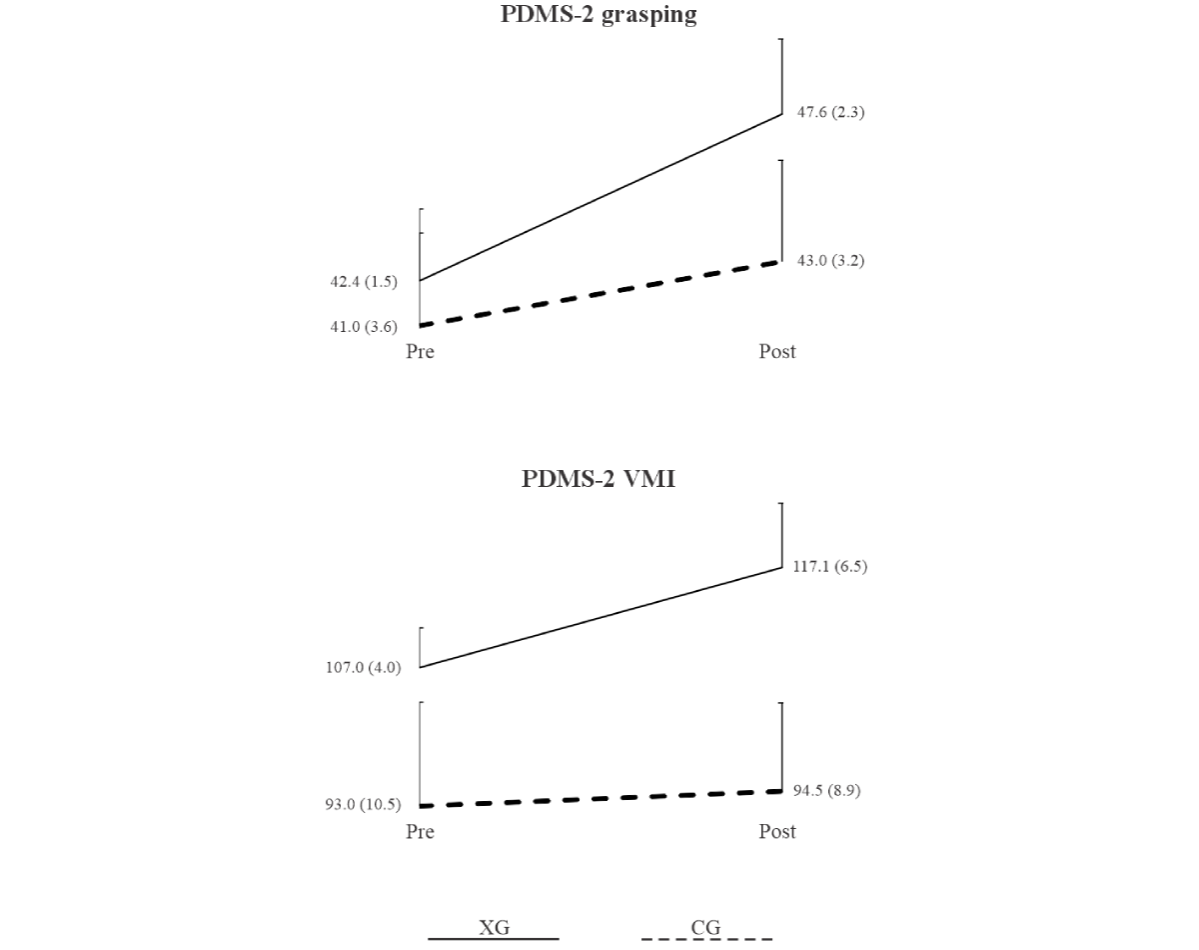
Group means and 95% CIs of PDMS-2 subtest scores before and after the intervention. Solid line indicates XG, and dashed line indicates CG. CG: control group; PDMS-2: Peabody Developmental Motor Scale-2; VMI: visual-motor integration; XG: experimental group.

Table 4 displays ANOVA results for the grasping and VMI subtest scores, examining time, group, and the time×group interaction effects. Significant effects of time, group, and the time×group interaction were observed for both PDMS-2 grasping and VMI test scores. Figure 5 depicts line plots of group means and 95% CIs before and after the intervention for these scores. It illustrates significantly greater improvements in PDMS-2 test scores for the XG compared to the CG, accompanied by moderate to large effect sizes indicating substantial improvements from pre- to postintervention assessments.

**Table 4:** ANOVA results for time, group, and the time×group interaction on Peabody Developmental Motor Scale-2 (PDMS-2) subtest scores.

PDMS-2 subtests	Time of the assessment (within-group)				XG^a^ vs CG^b^ (between-group)				Time×group		
	*F* test (*df*=1)	*P* value	η^2^	*F* test (*df*=1)		*P* value	η^2^	*F* test (*df*=1)		*P* value	η^2^
Grasping	132.26	<.001	0.81	6.51		.02	0.17	11.87		.002	0.28
VMI^c^	43.68	<.001	0.59	26.31		<.001	0.46	7.28		0.01	0.19

^a^XG: experimental group.

^b^CG: control group.

^c^VMI: visual-motor integration.

### Qualitative Analysis

#### Overview

In total, 10 parents and children from the XG agreed to participate in interviews following the 8-week intervention. The following 5 themes captured the range of parents’ and children’s experiences and opinions about their child’s exercise program. Textboxes 1-5 present parents’ direct quotes for each theme.

**Parent quotes on issues encountered.**

“We didn’t face any type of problem during the therapy, although my child had faced little challenging at first while playing the games, he didn’t understand the idea of gaming, but later he got to know how to play properly” [Parent 1].

“Some games involved one more person to assist the game as in to release the ball or bird to start the game, if even simpler games were included in treatment where child can only start the game and play easily would be really helpful” [Parent 5].

“Can improvise games, can add other interesting games specially cartoon ones which they watch regularly on TV, this will help them in playing the games even more interesting” [Parent 6].

“There were no serious problems faced, but my child had little issues with holding the handles properly, he had no idea about how exactly to hold the handles to move them to play. The main device keeps sliding a little while playing, so one person had to always hold the main device so that child can play without any problem, if the device can be fixed in one place or with better grip below it would be nice” [Parent 7].

“My child had difficulty in understanding what movement she should perform to play the games, such as catching the fish, she had little confusion with moving the handles left and right” [Parent 8].

“My child had difficulty in understanding the rules of the game” [Parent 9].

**Parent quotes on expectations for therapy outcomes.**

“I thought that my child will be able to do hand movements better and she will get better” [Parent 3].

“I hope that his concentration, eye hand co-ordination may improve with this therapy” [Parent 4].

“I thought that my child’s hand movements will improve also concentration will improve which will help him to use laptop” [Parent 5].

“I thought that my child’s mind will get sharp, she can see properly with her eyes while playing” [Parent 6].

“I thought that my child’s hand will get stronger with the help of this therapy” [Parent 7].

“Initially I have not predicted that this kind of therapy could help the child as I have no idea about this computer based therapy but later on I realized its very nice as it is based on game as well as work on multiple approaches in improvement by hand movement with eye co-ordination” [Parent 9].

“I thought because of this game therapy my child’s concentration would improve” [Parent 10].

**Parent quotes on motivation and engagement with the computer game–based protocol.**

“My child cries a lot while doing exercises, she would come to therapy after saying that she will be allowed to play computer games, because of this her therapy also used to happen. This I liked very much” [Parent 3].

“It’s more interesting for children, my child is doing very interestingly and happily, it helps to improve concentration and eye- hand co-ordination. My child was playing happily, he likes the fish game most” [Parent 4].

“My child used to do other exercises fast while thinking that he is playing games which I liked and, he was playing games very enthusiastically” [Parent 5].

“If she plays computer games, her mind will get sharp. She got to know that what fish, ball birds look like, and she used to play well with happiness” [Parent 6].

“I liked that because of this computer game therapy my child’s hand improved, she started using her hands for doing activities like to draw lines, coloring etc., which I liked about this therapy so much. She played well, she started to learn to play better later, and my child played all the games well” [Parent 7].

**Parent quotes on improvements in hand function and eye-hand coordination.**

“I feel technology is useful. It will help to improve my son’s hand problems and I liked it” [Parent 1].

“My child was not watching TV much, so I thought because of this computer games she might develop interest in watching TV and that her hand will gain strength and get better” [Parent 3].

“I liked that because of this computer game therapy my child’s hand improved, she started using her hands for doing activities like to draw lines, coloring etc., which I liked about this therapy so much” [Parent 7].

“At first my child was feeling very difficult to play the games, later he improved better, hence I was satisfied. Now he is using his hand better than before” [Parent 8].

“This therapy program basically improves the eye co-ordination with hand movement. It is nice therapy as it is done in game manner” [Parent 9].

“I thought because this game my child’s hand movement will improve this I liked very much. we can implement this technology based treatment for children, now my child is making his hand straight while playing games” [Parent 10].

**Parent quotes on suggestions for future implementation of computer game–based therapy.**

“If we could make them play for more time it would benefit them even more, this treatment should be added to regular exercise regimen” [Parent 1].

“My child had difficulty in understanding the speed and technique of the game at first and was not playing that well at first, which was the difficulty” [Parent 2].

“Some games involved one more person to assist the game as in to release the ball or bird to start the game, if even simpler games were included in treatment where child can only start the game and play easily would be really helpful” [Parent 3].

“Can improvise games, can add other interesting games specially cartoon ones which they watch regularly on TV, this will help them in playing the games even more interesting” [Parent 4].

“Kids who need this kind of treatment will be benefitted well, hence this treatment should be available to as many children as possible” [Parent 5].

“My child enjoyed playing games, but when my child was not well or due to some other issues when we were not able to go to therapy problem, my child would really miss this treatment, so if this game device was available for purchase it would really help” [Parent 6].

“If it could play for a greater number of times it would be even more helpful” [Parent 7].

“My child had difficulty in understanding the rules of the game” [Parent 8].

#### Issues

Parents found the computer game–based platform promising; however, they noticed that children initially found it hard to understand how the RMD device works and how to play games while using it. The RMD allows only 1 degree of freedom in any movement being practiced. The protocols for children were planned to repetitively practice challenging movements while performing a goal-directed task.

#### Parents’ Expectations

All parents participated in the study with the expectation of improvement in hand function for their child. Most parents also expected improvements in eye-hand coordination and better concentration. Due to socioeconomic barriers, 2 parents also hoped that their child would get better exposure to computers. A few parents hoped that the computer game–based protocol would motivate their child to stay focused during therapy sessions.

#### Motivation

All 10 parents and children found the computer game–based protocol engaging, challenging, and extremely motivating. All 10 parents would like the computer game–based protocol to continue as regular therapy and suggested that the games should be incorporated in more ways during therapy. Children responded well to the types of games chosen for their individualized protocols; however, it was necessary to use a wide variety of games to maintain their interest in the protocol.

#### Improvements in Hand Function and Eye-Hand Coordination

All 10 parents observed improvements in their child’s hand function and eye-hand coordination, consistent with the quantitative data. Many parents also noticed positive changes in their child’s overall level of participation in daily activities and their ability to concentrate on tasks.

#### Plans for Future

Parents observed that there was difficulty in the initial stages of their child’s participation and understanding in the computer game–based therapy program. However, after crossing the learning curve, children found the protocol engaging and fun. They suggested that this protocol should be available more frequently in clinical settings or at home to facilitate continuous improvement.

## Discussion

### Principal Findings

This study demonstrated the feasibility and therapeutic benefits of a game-based RMD for improving manual dexterity in children with CP. Both the XG and the CG showed significant improvements in the PDMS-2 subtests and the CUE assessment of manual dexterity. In addition, the XG outperformed the CG, with greater improvements in PDMS-2 grasping and VMI as well as higher success rates and fewer movement errors in the CUE assessment. Additionally, parents reported an increase in their children’s independence in daily tasks, reflecting the positive impact of the game-based exercise program on functional hand use.

The RMD’s design and functionality are crucial to achieving the observed therapeutic benefits. Various handles of different sizes and shapes were used, which could be handled with a 2-finger grip, 3-finger grip, or whole-hand grasp, and manipulated during gameplay using thumb-finger motion, wrist motion, or a combination of elbow and shoulder motions. Both the types of handle motions and the choice of computer games have specific therapeutic value. Many inexpensive commercial computer games that can be played with the RMD require different levels of movement amplitude and precision, that is, speed and accuracy. Regular introduction of new games and progression through difficulty levels are crucial to maintain the interest and participation of the children.

Several researchers and clinicians emphasize the importance of high repetition and task-specific training in facilitating the recovery of hand function [12,51,52]. One main feature of the RMD game–assisted exercise program was to increase the number of repetitions of goal-directed movements, that is, graded speed and accuracy. Each game was played for 2-3 minutes, with each game event lasting approximately 2 seconds, resulting in 60-90 goal-directed game movement responses per game. Typically, each child played several computer games for 30 minutes. Thus, during each exercise session, the children made several hundred game movement responses. This high number of repetitions involved goal-directed, precision movements with random directions, variable amplitudes, and speeds. Additionally, visual feedback of the game paddle relative to various moving game objects was always available. Continuous visual feedback guided the position and motion of the game paddle or sprite rather than the object being manipulated. This type of practice promotes implicit learning of eye-hand coordination [53,54].

Initially, children with severe impairments succeeded in games that involved slow movements and low precision, such as large paddle sizes, large game target objects, few distractor objects, and limited cognitive demands. Children with moderate to mild impairment could engage in a larger variety of games that had faster movement speeds, greater movement precision, and added cognitive demands.

There are no published reports that have calculated the minimal clinically important difference (MCID) for either grasping or VMI subtests. However, the MCID for the total motor quotient has been reported to be 8.3% [55]. In this study, the percentage improvement in grasping and VMI subtests for the XG was 12% and 9.3%, respectively, exceeding the MCID reported for the total motor quotient. The improvements observed for the XG were significantly greater than those observed for the CG, that is, 4% for grasping and 2% for VMI. In the previous study [3], a randomized clinical trial evaluated the effects of a game-based exercise program using an IB computer mouse on manual dexterity in young children with CP aged 4-10 years. The CG received task-specific training similar to that used in constraint-induced movement therapy. Each group received 16 weeks of therapy, 3 times per week. They observed an 18% improvement in PDMS-2 grasping and a 12% improvement in PDMS-2 VMI for the group that received the game-based exercise program. Improvement in the CG was 12% for PDMS-2 grasping and 9% for PDMS-2 VMI. In the previous study [3], the intervention lasted 16 weeks, twice as long as the duration of this study. This discrepancy likely explains the variation in percentage improvement observed in grasping and VMI between the 2 studies: 18% versus 12% for PDMS-2 grasping and 12% versus 9.3% for PDMS-2 VMI.

The PDMS-2 grasping measures daily activities involving the fingers of both hands, such as picking up small objects, buttoning and unbuttoning, stringing beads, and using hand tools. Significant improvements in PDMS-2 grasping were observed despite these tasks not being practiced during the game-based manipulation exercise program. The PDMS-2 VMI assesses an individual’s ability to use visual perceptual skills for various eye-hand coordination tasks, including stacking blocks and drawing figures. In this study, the RMD game tasks required precise movements based on visual feedback from the moving game objects. This requirement is reflected in the substantial improvements seen in both the PDMS-2 tasks and the CUE object manipulation tasks.

Most parents expressed their willingness to join the trial because therapy focused on manual dexterity and addressed the lack of eye-hand coordination in their children. Both the XG and CG showed significant improvements in success rate and a decrease in movement error in all 4 object manipulation tasks, with moderate to large effect sizes. There was a significant time×group interaction, demonstrating that improvements in success rate were significantly greater for XG compared to CG.

The improvements observed in the CUE assessment of function, as well as in both the PDMS-2 grasping and VMI, were consistent with the quantitative data. Parents reported positive changes in their child’s hand function, eye-hand coordination, overall level of participation in daily activities, and concentration levels. They agreed that the addition of computer games was motivating and encouraged their children to actively participate and have fun during exercise sessions. Most parents from the XG commented that the game-based exercises were challenging yet engaging and that their children enjoyed playing them. During the initial stages of the protocol, some parents observed reluctance due to a lack of understanding of the computer games and difficulty with producing precision movements. However, after 2-3 sessions, most parents felt that the computer game–based platform provided a promising way to improve children’s compliance with the therapy program. Other studies incorporating computer games in therapy have also reported that children enjoyed the games used in their exercise programs [13,15,34,35].

### Future Enhancements

The unidirectional force mode, while assisting movement in 1 direction, does not ensure that the handle movements will successfully interact with the target game objects. Additionally, 1 movement direction may receive resistive force, which may not be desirable. A real-time intelligent control scheme is under development, involving communication between the manipulandum and the RTP game. The controller will receive the coordinates of both the game targets and the game paddle. This knowledge about movement directions and amplitudes will be used to determine the direction and magnitude of the force required to rotate the handle and assist the subject in moving the game paddle as needed to interact effectively with the game target objects. This closed-loop assistance can be provided in both movement directions during gameplay, amplifying limited and small voluntary movements of those severely affected while allowing opportunities for progression with increased movement demands.

It is also common for children with paralysis to experience sudden, uncontrolled movements and difficulty managing their strength. To address this, a design change was made after the study to allow the device to be securely attached to a table, preventing unwanted movement during use. The modified device can now be easily attached to a table, providing greater stability and control during gameplay.

### Conclusions

The results of this feasibility study demonstrate the feasibility, acceptability, and positive outcomes of the RMD game–based exercise program for the rehabilitation of hand function in children with CP. The findings indicate that computer game–assisted exercise programs can improve manual dexterity in children with CP. The results support further research and development related to the effects of computer game–assisted rehabilitation technology. The long-term effects of computer game–based training on hand function will need to be confirmed in future RCTs.

## Appendix

Multimedia Appendix 1 CONSORT (Consolidated Standards of Reporting Trials)-EHEALTH V1.6 checklist.

Multimedia Appendix 2 Example computer games used in the experimental exercise program.

## Abbreviations

CG: control group
CONSORT: Consolidated Standards of Reporting Trials
CONSORT-EHEALTH: Consolidated Standards of Reporting Trials of Electronic and Mobile Health Applications and Online Telehealth
CP: cerebral palsy
CUE: computer game–based upper extremity
GMFCS: Gross Motor Function Classification System
IB: inertial-based
MACS: Manual Ability Classification System
MCID: minimal clinically important difference
PDMS-2: Peabody Developmental Motor Scale-2
RCT: randomized controlled trial
RMD: robotic manipulandum device
RTP: repetitive task practice
VMI: visual-motor integration
XG: experimental group
