# Supplementary material for: Evaluation of a Game-Based Mechatronic Device for Rehabilitation of Hand-Arm Function in Children With Cerebral Palsy: Feasibility Randomized Controlled Trial
Source: JMIR Rehabil Assist Technol. 2025 Feb 18;12:e65358. doi: 10.2196/65358 (PMC11888099; doi:10.2196/65358)
Supplement: Multimedia Appendix 2 [file rehab_v12i1e65358_app2.docx]

| **Game Title** | **Type / Activity** | **Axis Play** | **Start Difficulty** | **Response Time** | **Precision** | **Distractor** |
| --- | --- | --- | --- | --- | --- | --- |
| Abundate | Matching (Select and Group) | Horizontal | Moderate | Moderate | High | No |
| Action ball | Brick buster | Horizontal | Moderate | Fast | Moderate | Yes |
| Aqua Ball | Brick buster | Horizontal | Easy | Slow | Moderate | Yes |
| Astrobugs Revenge | Matching | Horizontal | Moderate | Slow | High | No |
| Birds Town | Matching | Horizontal | Moderate | Moderate | High | No |
| Brave Piglet | Shooting | Vertical | Easy | Moderate | Low | Yes |
| Butterfly Escape | Matching | Horizontal | Moderate | Moderate | High | No |
| Bubble Town | Matching | Horizontal | Easy | Slow | High | No |
| Cubis Creatures | Matching | Horizontal | Easy | Moderate | Low | No |
| Deepica | Matching | Horizontal | Easy | Slow | Low | No |
| Digby Donuts | Matching (Catch & Group) | Horiztonal | Moderate | Moderate | High | No |
| Egyptian Ball | Brick buster | Horizontal | Moderate | Moderate | Moderate | Yes |
| Fishing Craze | Fishing | Horizontal | Moderate | Moderate | Low | Yes |
| Hyperballoid 2 | Brick buster | Horizontal | Moderate | Fast | Moderate | Yes |
| Jar of Marbles | Matching | Horizontal | Easy | Slow | High | No |
| Jet Jumper | Driving | Horizontal | Difficult | Fast | High | Yes |
| Luxor 3 | Matching | Horizontal | Moderate | Moderate | High | No |
| Ozzy bubbles | Platformer | Horizontal | Easy | Slow | Low | Yes |
| Reaxxion | Brick buster | Horizontal | Moderate | Fast | Moderate | Yes |
| Stoneloops! Of Jurassica | Matching (Select and Group) | Horizontal | Difficult | Fast | High | No |
